# Overground exoskeletons may boost neuroplasticity in myotonic dystrophy type 1 rehabilitation: A case report

**DOI:** 10.1097/MD.0000000000017582

**Published:** 2019-11-15

**Authors:** Simona Portaro, Antonino Naro, Antonino Leo, Vincenzo Cimino, Tina Balletta, Antonio Buda, Maria Accorinti, Rocco Salvatore Calabrò

**Affiliations:** Neurorobotic and Rare Disease Unit, IRCCS Centro Neurolesi “Bonino Pulejo”, Messina, Italy.

**Keywords:** Ekso, myotonic dystrophy type 1, overground exoskeleton, robotic training, sensory-motor integration

## Abstract

**Rationale:**

Myotonic dystrophy type 1 (DM1) is a slowly progressive multisystem neuromuscular disease characterized by myotonia and muscle weakness and wasting of distal and axial muscles. People with DM1, due to the disease progression, are often concerned about their ability to carry out and participate in the activities of daily living. Rehabilitation approaches in DM1, including moderate-to-intense strength training, have shown not univocal efficacy to face such difficulties. Aim of this case-study was to demonstrate the effects of a combined approach by using conventional plus robotic training in rare neuromuscular diseases, such as DM1.

**Patient concerns:**

A 46-year-old woman came to our observation complaining of difficulty in opening fist after strong voluntary muscle contraction for about 20 years. Over the years, she referred swallowing difficulties for solid foods, balance impairment complicated by tendency to stumble and falls, fatigability, hand muscle weakness with difficulty to open bottles and lifting weights, and daytime sleepiness

**Diagnosis:**

Paraparesis in DM1.

**Interventions:**

The patient underwent 2 different trainings. The first period of treatment was carried out by using conventional physiotherapy, 6 times a week (twice a day) for 4 weeks. Then, she underwent a two-month specific task-oriented robotic rehabilitation training for the gait impairment using an overground exoskeleton, namely Ekso-GT, combined to the conventional therapy.

**Outcomes:**

The patient, after the EKSO training, gained a significant improvement in walking, balance and lower limbs muscle strength, as per 10-meter walking test and Left Lower Limb Motricity Index. Neurophysiological data (electroencephalography and surface electromyography) were also collected to more objectively assess the functional outcomes.

**Lessons:**

Rehabilitation approaches in DM1, including moderate-to-intense strength training, have shown not univocal efficacy. Emerging and advancing robotic technologies can enhance clinical therapeutic outcomes by allowing therapists to activate and/or modulate neural networks to maximize motor and functional recovery.

## 1 Introduction

Myotonic dystrophy type 1 (DM1) is a slowly progressive neuromuscular disease characterized by myotonia and muscle weakness and wasting of distal and axial muscles, thus causing gait and balance disturbances. People with DM1, due to the disease progression, are often concerned about their ability to carry out and participate in the activities of daily living (ADL). Rehabilitation approaches in DM1, including moderate-to-intense strength training, have shown not univocal efficacy to face such difficulties.^[1]^ Nevertheless, hand-training programs aimed at containing distal muscle weakness seems to be effective to improve hand motor function and ADL performances.^[2]^ Non-conventional training programs, such as tap dance, have been anecdotally used to improve static and dynamic balance in the early stage of the disease.^[3]^ Neurorobotic devices have been increasingly used to improve motor outcomes in a wide variety of neurologic conditions, with particular regard to stroke.^[4,5]^ However, there are no data regarding their application in DM1 patients, even though a few reports showed some positive effects of robotic devices in neuromuscular diseases.^[6]^ Moreover, there is growing evidence on the usefulness of harnessing neuroplasticity mechanisms to improve function recovery by realizing patient-tailored rehabilitative programs (i.e., based on individual neuroplasticity potential to recovery). Indeed, it has been recently shown the potential role of neuroplasticity mechanisms in improving motor outcome in patients with DM1.^[7]^

Herein, we report a case of a 46-year-old woman with DM1, who presented an important functional recovery only after a robotic rehabilitation training by the means of Ekso, an exoskeleton framework for the lower limbs.

## 2 Case report

A 46-year-old woman came to our observation complaining of difficulty in opening fist after strong voluntary muscle contraction for about 20 years. Over the years, she referred swallowing difficulties for solid foods, balance impairment complicated by tendency to stumble and falls, fatigability, hand muscle weakness with difficulty to open bottles and lifting weights, and daytime sleepiness. At neurological examination, she presented bilateral palpebral ptosis and facial muscle weakness, waddling gait with bilateral steppage, which was not possible on tiptoes and heels, axial muscle weakness, including neck flexor muscles. Left pectoral muscle, wrist and fingers flexor and extensor muscles, interosseous muscles, anterior tibialis were also weak and hypotrophic. Deep tendon reflexes were absent. Myotonic phenomenon was present in the hands, tongue, and jaw. Slight reduction in range of motion of the left ankle was also evident. ADL performance (Barthel index 60/100) were moderately affected. She did not present thyroid and metabolic diseases. Cardiological screening disclosed left anterior bundle hemi-block without clinical significance. Pneumological assessment showed a restrictive respiratory pattern, with no need for non-invasive mechanical ventilation.

Patient gave written informed consent for the diagnostic procedures, treatment, and publication of the case.

### 2.1 Intervention

She then started a physiotherapy program. The first period of treatment was carried out by using conventional physiotherapy, in inpatient regimen, 6 times a week (twice a day) for 4 weeks, each session lasting at least 50 minutes. However, after such training, the patient did not gain any significant functional recovery, so she stopped the rehabilitation treatment for about 1 month. Then, she underwent a two-month specific task-oriented robotic rehabilitation training for the gait impairment using an overground exoskeleton, namely Ekso-GT, combined to the conventional therapy.

The Ekso protocol session was scheduled five times a week for 8 consecutive weeks (for 40 sessions). Ekso is an exoskeleton framework for the lower limbs, equipped with

**Figure 1:**
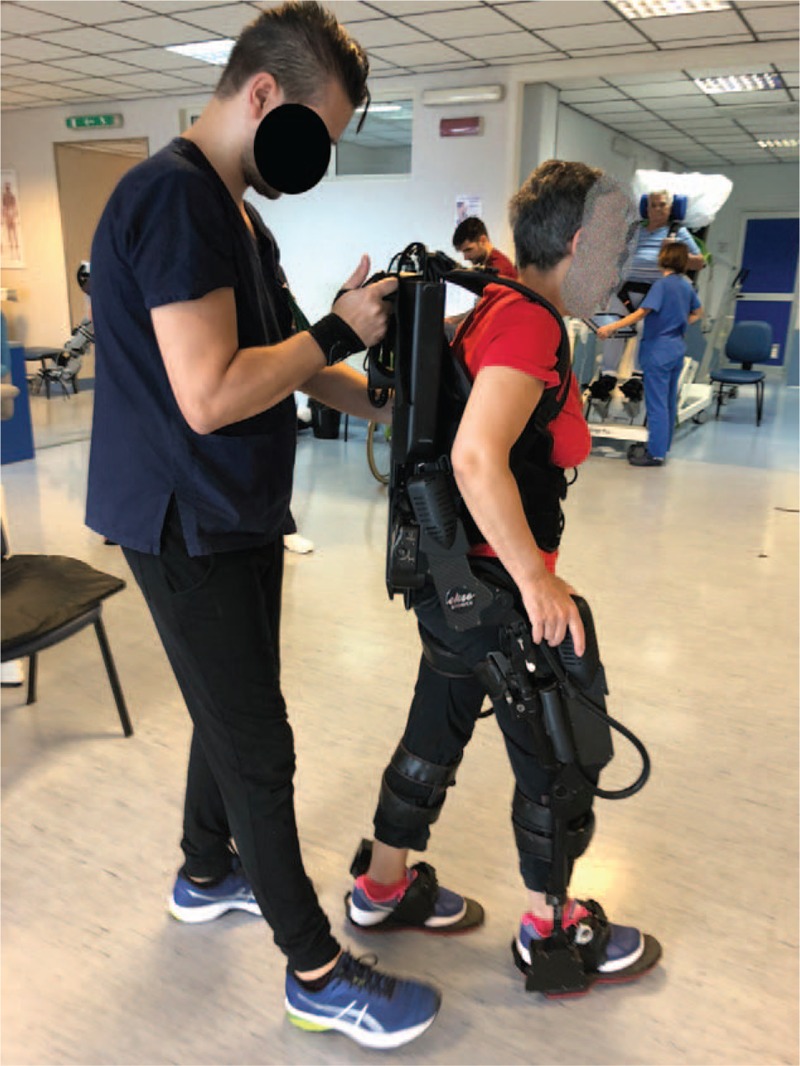
shows a typical rehabilitation training with the overground exoskeleton Ekso-GT.

(1) electric motors to power movement for the hip and knee joints,
(2) passive spring-loaded ankle joints,
(3) foot plates on which the user stands, and
(4) a backpack that houses a computer, battery supply, and wired controller (Fig. 1).

The limb and pelvic segments are adjustable to the user's leg and thigh length, and the segment across the pelvis is adjustable for hip width and hip abduction angle. Once set up the exoskeleton on patient's physical characteristics (e.g., physical size, lower limb measures), under an Ekso-trained physiotherapist supervision, the patient started the specific robotic training, slowly adapted to her compliance. The conventional rehabilitation (each session lasting about 30 minutes and preceding the robotic training) consisted of bilateral muscle stretching, muscle strength training, gait and balance training.

### 2.2 Outcomes

Outcome measures were the 10-meter walking test (10MWT), the Motricity Index (MI), the timed up-and-go test (TUG) and BERG balance scale. These were administered at baseline (T0), after the end of the first period of conventional treatment (T1), before (T2) and after (T3) the combined conventional and robotic rehabilitation protocol. Moreover, EEG and gait analysis were performed at T2 and T3. The primary goal was to obtain a significant improvement in lower limb gait and balance (as per the Reliable change index, RCI) at the end of the combined conventional and robotic rehabilitation protocol.

### 2.3 Neuroplasticity evaluation

In addition, the EEG recording and analysis was also performed. Specifically, EEG was recorded using a high-input impedance amplifier Brain Quick System-PLUS (Micromed; Mogliano Veneto, Italy), wired to a standard 21-electrode headset. An electro-oculogram (0.3–70 Hz band-pass) was also recorded. The recording occurred in the morning (about 11am) and lasted at least 10 minutes, with the eyes open (fixing a point in front of the patient). The EEG end electro-oculogram were sampled at 512 Hz, filtered at 0.3 to 70 Hz, and referenced to linked earlobes.^[8]^

EEG recordings contaminated by blinking, eye movements, movements, and other artifacts were rejected off-line by visual inspection and based on independent component analysis (ICA) data. First, we identified the cortical activations induced by gait training from the EEG recordings by using Low-Resolution Brain Electromagnetic Tomography (LORETA; free release of LORETA-KEY alpha-software).^[9–13]^ The brain compartment of the 3-shell spherical head model used in the LORETA was restricted to the cortical gray matter, Talairach co-registered,^[14]^ and had a resolution of 7 mm, thus obtaining 2394 voxels (i.e., equivalent current dipoles). The voxels of LORETA solutions were collapsed in seven regions of interest (prefrontal, supplementary motor, centro-parietal, and occipital areas of both hemispheres)^[15]^ determined according to the brain model coded into Talairach space. Then, structural equation modeling (SEM) technique (or path analysis) was employed to measure the effective connectivity (that assesses the causal influence that one brain area, that is, electrode-group, exerts over another, under the assumption of a given mechanistic model)^[16,17]^ among the cortical activations induced by gait trainings. SEM combines a network model supporting the putative connections linking sets of cortical activations and the inter-regional covariances of activity (i.e., the degree to which the activities of 2 or more regions, i.e., electrode-group, are related), to estimate the influence of one region (electrode set) on another through the putative connections linking the sets of (electrode) activation.^[15]^ The network model, supporting inter-regional connectivity employed in our study, was defined according to a previous study and included the abovementioned regions of interest.^[15]^

### 2.4 Gait analysis

Gait data analysis was performed using an eight-channel wireless sEMG device (BTS; Milan, Italy) and recording the EMG activity (sampled at 1-kHz, filtered at 5-300 Hz) from eight muscles (both tibialis anterior, soleus, rectus femoris, and biceps femoris). The EMG signal was analyzed for root-mean-square (a temporal parameter estimating muscle activation) to investigate lower-limb muscle activation modified by gait training.^[18]^

The device was also equipped with an accelerometer put at lumbar level to establish the gait phases. Gait analysis was conducted on a 10-meter walkway and 2 gaits at a self-selected speed were collected. We measured the gait quality index before and at the completion of the gait training. This is an overall gait performance score reflecting an approximate 60%:40% distribution of stance:swing phases (ratio between stance from heel strike to toe-off, and swing phase duration from toe-off to heel strike), a normal step cadence, and a regular gait cycle duration (time from 1 right heel strike -initial contact- to the next one -end of terminal swing). Gait quality index values were averaged from the 2 runs and used for subsequent analyses.

## 3 Results

**Figure 2:**
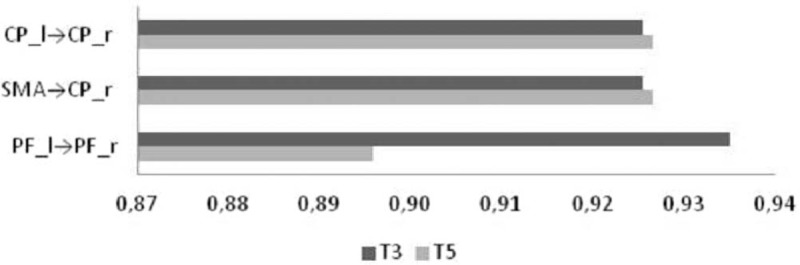
shows the modulation of functional connectivity after the lower limb robotic training. CP = centro-parietal (area), PF = prefrontal (area), SMA = sensory-motor area.

The patient performed the robotic training without any side effect. Only at the end of the experimental training, we found a significant improvement in BERG score, TUG e Left Lower Limb MI (see Table 1). Notably, the patient showed a significant improvement in Gait quality index, from 79% to 88% (RCI = –6.36), which depended on the improvements in step cadence (from 1.2 to 1.6 Hz, RCI = –2.83), gait cycle duration (from 1.8 to 1.4s RCI = 2.98), and stance:swing ratio (from 72:28 to 63:37%; RCI = 4.46). There was also a significant improvement in muscle activation. In particular, the coupled approach increased the hip extension during the stance phase and the ankle power generation in terminal stance. On the other hand, it limited the knee hyperextension during the mid-stance, the high dorsiflexion during the stance phase, and the high hip power during loading response. The findings were paralleled by a significant modulation of functional connectivity (*time* *×* *path* F_(1,20)_ = 5.6, *P* = .03, η^2^ = 0.6), with particular regard to a connectivity strengthening of Prefrontal_l→Prefrontal_r, and a reduction of Sensory-motor→Centro-Parietal_r, and Centro-parietal_l→ Centro-parietal_r areas (Fig. 2).

**Table 1:**
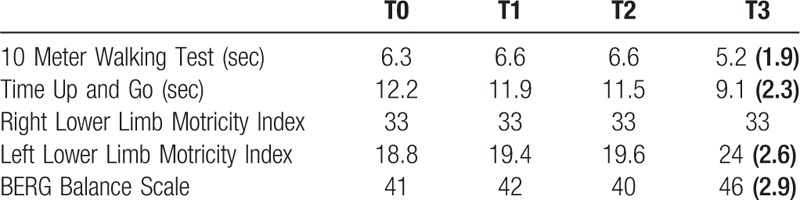
shows the main clinical outcomes after each training with the correlated Reliable Change Index in ().

## 4 Discussion

To date, few data exist on the efficacy of rehabilitation training in neuromuscular disorders. Moreover, scarce evidence are reported on the use of robotic devices in such diseases.^[2,3,6]^ Physiotherapy, including muscle stretching, balance and gait training and occupational therapy involving fatigue management and functional retraining in tasks of daily living, may reduce muscle atrophy and improve endurance, as regular exercise may attenuate the aging process by potentiating the cardiovascular, respiratory, endocrine, musculoskeletal, and immune systems. To this end, a recent study carried out on ALS patients^[19]^ showed that a strictly monitored exercise program might significantly reduce motor deterioration. Robotic assisted gait training may be considered a valuable tool to improve muscle force and slow neuromuscular deterioration thanks to the possibility to administer aerobic exercises in a controlled manner. Indeed, the efficacy of neurorobotics in improving motor function depends on the high frequency and intensity of repetition of task-oriented movements, resulting in motor learning.^[20]^

Moreover, emerging and advancing robotic technologies can enhance clinical therapeutic techniques by allowing therapists to activate and/or modulate neural networks to maximize recovery and functional outcomes.^[21]^ In particular, with the addition of robotic exoskeletons, therapists are now able to safely, and more efficiently get patients with lower extremity weakness or paralysis to perform higher frequency of robotic-assisted stepping that resembles normal stepping pattern than achievable through more traditional means of gait training. To date, robotic exoskeletons have been primarily used in clinical settings as an adjunct tool to customary rehabilitative treatment approaches, with regard to gait training or to mobilize low functioning patients with different neurological disorders. Exoskeletons can be utilized to provide a walking experience for nonfunctional ambulatory individuals, as an early intervention strategy for neuromuscular reconditioning and gait retraining among very acutely injured and patients with minimal functional mobility, or as a gait retraining tool with more chronically injured and high functioning ambulators. Even acutely injured patients with little or no functional mobility can benefit from the use of exoskeletons to promote head and trunk control in a dynamic standing and weight-bearing position, in order to reconditionate sensory-motor functions. Sensorimotor control is a complex process involving the integration of the information provided by the sensory system on the conditions of joints, muscles, and skin by a network of neurons, interneurons, and cortical networks to provide coordinated voluntary movements. Several studies have highlighted the important cortical activities during gait and balance.^[22]^ As shown in a previous study, exoskeletons could be useful to recovery mobility in people with neurological impairment due to central nervous system diseases, that is, stroke, owing to mechanisms of brain plasticity and connectivity re-modulation that are specifically entrained by the robotic device, as compared to conventional physiotherapy.^[23]^ Thus, rehabilitation techniques targeting sensory-motor systems could be beneficial for improving gait capacity in patients with neurological disorders, including neuromuscular disorder. Indeed, it has been shown the potential role of neuroplasticity mechanisms in improving motor outcome in patients with DM1.^[7]^ On such basis, for the ever first time, we decided to submit to robotic rehab (i.e. overground exoskeletons) a patient affected by DM1. In these patients, muscle weakness leads to long-term deconditioning of sensorimotor function in the affected limbs, thus worsening ambulatory pattern. Own personal motor ability, derived from personal experience, could contribute to determine specific functional reserve, influencing the maintenance of bone mass, balance, sleep, bowel and bladder functioning, reducing pain and fatigue, improving overall body conditioning. As those benefits, derived from functional reserve, are certainly important for improving long-term adaptation associated to DM1 progression, use of robotic devices as a clinical treatment tool can be helpful to maintain it, above all do to its capacity to influence sensory motor plasticity, in order to preserve walking independence on DM patients.

In our case, we showed a significant improvement in walking, balance and lower limbs muscle strength, gained only after the combined approach (i.e. conventional plus robotic training). We can postulate that sensory-motor integration, together with a repetitive, task-oriented and intense rehabilitation robotic training may play a comprehensive role on neuroplasticity, although DM1 is not a central nervous system disorder, but a muscle primary chronic degenerative disease.

However, even though exoskeletons may improve musculoskeletal and neuromuscular performances, there are significant limitations for their application, that is, device weight, portability and assembly issues, the need for upper extremity supports, supervision requirements, and the limited range of movements, thus confining the use of such devices to neurorehabilitation units with experience in robotics.

We are aware that findings from a single case report have many limitations, including epidemiological bias, impossibility of causal inference and generalization and over-interpretation. Further studies are needed to support these promising findings, also taking into account the long- term effect of neurorobotics in neuromuscular disorders.

## Author contributions

**Conceptualization:** Simona Portaro, Antonino Naro, Antonio Buda, Rocco Salvatore Calabrò.

**Data curation:** Simona Portaro, Antonino Naro, Antonino Leo, Vincenzo Cimino, Tina Balletta, Antonio Buda, Maria Accorinti.

**Investigation:** Simona Portaro, Antonino Leo, Vincenzo Cimino, Tina Balletta, Antonio Buda, Maria Accorinti.

**Methodology:** Antonino Naro, Antonino Leo, Tina Balletta, Antonio Buda, Maria Accorinti, Rocco Salvatore Calabrò.

**Resources:** Vincenzo Cimino.

**Supervision:** Rocco Salvatore Calabrò.

**Visualization:** Antonio Buda, Maria Accorinti.

**Writing – original draft:** Simona Portaro.

**Writing – review & editing:** Antonino Naro, Rocco Salvatore Calabrò.

Rocco Salvatore Calabrò: 0000-0002-8566-3166
